# Ethyl 2-[(*E*)-4-(dimethyl­amino)benzyl­idenehydrazino]-5-nitro­benzoate

**DOI:** 10.1107/S1600536808035939

**Published:** 2008-11-08

**Authors:** Hoong-Kun Fun, Adithya Adhikari, P. S. Patil, B. Kalluraya, Suchada Chantrapromma

**Affiliations:** a X-ray Crystallography Unit, School of Physics, Universiti Sains Malaysia, 11800 USM, Penang, Malaysia; b Department of Studies in Chemistry, Mangalore University, Mangalagangotri, Mangalore 574 199, India; c Department of Physics, K.L.E. Society’s K.L.E. Institute of Technology, Gokul Road, Hubli 590 030, India; d Crystal Materials Research Unit, Department of Chemistry, Faculty of Science, Prince of Songkla University, Hat-Yai, Songkhla 90112, Thailand

## Abstract

The title compound, C_18_H_20_N_4_O_4_, exists in the *E* configuration with respect to the C=N bond of the methyl­idine unit. The dihedral angle between the two benzene rings is 9.01 (6)°. An intra­molecular N—H⋯O hydrogen bond involving the benzoate unit generates an *S*(6) ring motif. In the crystal, the mol­ecules are linked by weak C—H⋯O inter­actions into infinite chains along the *b* axis. These chains are further connected into sheets parallel to the *ab* plane which are stacked approximately along the *c* axis. A C—H⋯π inter­action is also observed.

## Related literature

For related literature on hydrogen-bond motifs, see: Bernstein *et al.* (1995 ▶). For bond-length data, see: Allen *et al.* (1987 ▶). For background to the applications of hydrazones, see, for example: Barton *et al.* (1962 ▶); Bedia *et al.* (2006 ▶); Buu-Hoi *et al.* (1953 ▶); Paquette (1995 ▶); Rollas *et al.* (2002 ▶); Terzioglu & Gürsoy (2003 ▶).
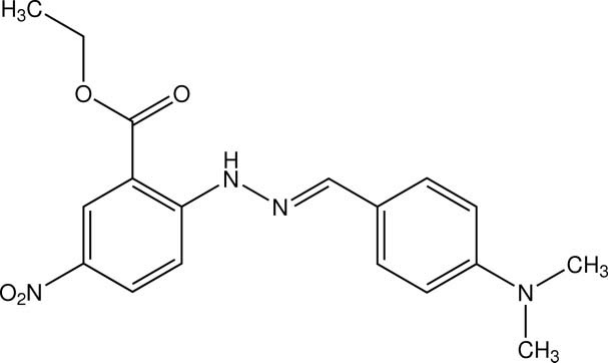


## Experimental

### 

#### Crystal data

C_18_H_20_N_4_O_4_
*M* *_r_* = 356.38
Monoclinic, 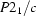

*a* = 10.8216 (4) Å
*b* = 15.9175 (6) Å
*c* = 10.4136 (4) Å
β = 107.091 (2)°
*V* = 1714.56 (11) Å^3^
*Z* = 4
Mo *K*α radiation
μ = 0.10 mm^−1^
*T* = 100.0 (1) K
0.44 × 0.41 × 0.31 mm

#### Data collection

Bruker SMART APEXII CCD area-detector diffractometer
Absorption correction: multi-scan (*SADABS*; Bruker, 2005 ▶) *T*_min_ = 0.957, *T*_max_ = 0.970
16368 measured reflections
3929 independent reflections
3275 reflections with *I* > 2σ(*I*)
*R*_int_ = 0.029

#### Refinement

*R*[*F* ^2^ > 2σ(*F* ^2^)] = 0.038
*wR*(*F* ^2^) = 0.105
*S* = 1.04
3929 reflections
242 parameters
H atoms treated by a mixture of independent and constrained refinement
Δρ_max_ = 0.26 e Å^−3^
Δρ_min_ = −0.27 e Å^−3^

### 

Data collection: *APEX2* (Bruker, 2005 ▶); cell refinement: *SAINT* (Bruker, 2005 ▶); data reduction: *SAINT*; program(s) used to solve structure: *SHELXTL* (Sheldrick, 2008 ▶); program(s) used to refine structure: *SHELXTL*; molecular graphics: *SHELXTL*; software used to prepare material for publication: *SHELXTL* and *PLATON* (Spek, 2003 ▶).

## Supplementary Material

Crystal structure: contains datablocks global, I. DOI: 10.1107/S1600536808035939/is2354sup1.cif

Structure factors: contains datablocks I. DOI: 10.1107/S1600536808035939/is2354Isup2.hkl

Additional supplementary materials:  crystallographic information; 3D view; checkCIF report

## Figures and Tables

**Table 1:** Hydrogen-bond geometry (Å, °)

*D*—H⋯*A*	*D*—H	H⋯*A*	*D*⋯*A*	*D*—H⋯*A*
N2—H1*N*2⋯O4	0.875 (18)	1.978 (17)	2.6736 (14)	135.6 (14)
C7—H7*A*⋯O1^i^	0.93	2.49	3.3599 (16)	156
C12—H12*A*⋯O4^ii^	0.93	2.59	3.3961 (16)	145
C16—H16*C*⋯O2^iii^	0.96	2.59	3.5116 (19)	162
C17—H17*B*⋯*Cg*1^iii^	0.96	2.64	3.4629 (14)	144

Symmetry codes: (i) 
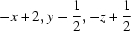
; (ii) 
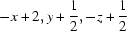
; (iii) 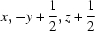. *Cg*1 is the centroid of the C1–C6 ring.

**Fig. 1.**
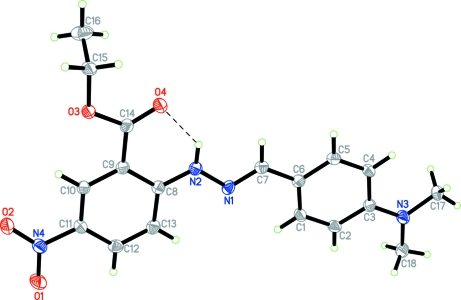
The asymmetric unit of (I), showing 50% probability displacement ellipsoids and the atomic numbering.

**Fig. 2.**
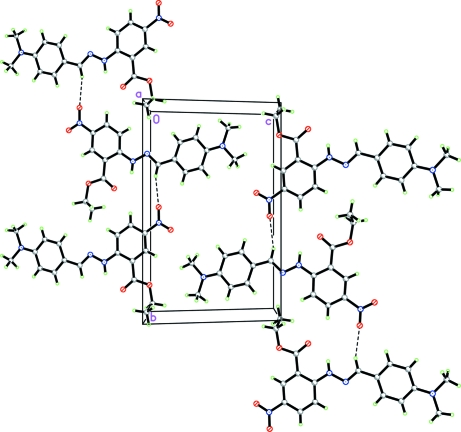
The crystal packing of (I), viewed along the a axis showing that the molecules are linked into infinite chains along the b axis. Hydrogen bonds are drawn as dashed lines.

**Fig. 3.**
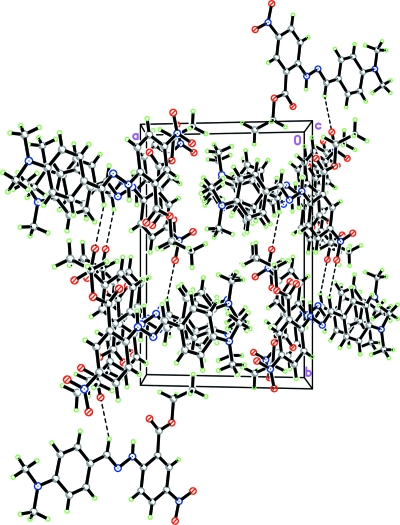
The crystal packing of (I), viewed approximately along the c axis. Hydrogen bonds are drawn as dashed lines.

## supplementary crystallographic information

### Comment

Hydrazine is widely used as a reagent in synthetic organic chemistry but is probably most frequently associated with the transformation of carbonyl-containing compounds to the corresponding hydrazones (Paquette, 1995). These are intermediates in the Wolff-Kishner reduction as well as many other reactions of synthetic utility, such as the Barton vinyl iodide preparation (Barton *et al.*, 1962). Hydrazones have been demonstrated to possess antimicrobial, anticonvulsant, analgesic, antiinflammatory, antiplatelet, antitubercular, anticancer and antitumoral activities (Bedia *et al.*, 2006; Rollas *et al.*, 2002; Terzioglu & Gürsoy, 2003). Hydrazones possessing an azometine –NHN=CH– proton constitute an important class of compounds for new drug development. Therefore, many researchers have synthesized these compounds as target structures to evaluate their biological activities. These observations have been the guides for the development of new hydrazones that possess varied biological activities. Some synthesized hydrazide-hydrazones were reported to have lower toxicity than hydrazides because of the blockage of –NH2 group (Buu-Hoi *et al.*, 1953). These findings further support the growing importance of the synthesis of hydrazide-hydrazones compounds.

Figure 1 shows the molecular structure of the title compound. The total molecule is not planar and exist in the *E* configuration with respect to the C═N bond of methylidine moiety. The dihedral angle between the two benzene rings is 9.01 (6)°. The methylidine is co-planar with the C1–C6 benzene ring [the most deviation of 0.044 (1) Å of atom C3] with the torsion angle N2–N1–C7–C6 = -179.18 (10)°. The dimethylamino group is slightly twisted from the plane C1–C6 ring as indicated by the torsions angle of C17–N3–C3–C4 = -6.57 (18)° and C18–N3–C3–C4 = -177.96 (12)°. The nitro group is slightly twisted from the C8–C13 benzene ring with the interplanar angle between the mean plane through N4/O1/O2/C11 and C8–C13 planes [8.17 (7)°]. The ethyl group is nearly perpendicularly attached to the benzoate unit which can be reflected by the torsion angle C14–O3–C15–C16 = 88.66 (13)°. An intramolecular N2—H1N2···O4 hydrogen bond generates an S(6) ring motif (Bernstein *et al.*, 1995) (Fig. 1 and Table 1). Bond lengths and angles in the title compound are in normal ranges (Allen *et al.*, 1987).

Figure 2 shows that the molecules are linked into infinite chains along the *b* axis through weak C7—H7A···O1 interaction (Table 1) and these chains are further connected through weak C—H···O interactions (Table 1) forming sheets parallel to the *ab* plane. These sheets are stacked approximately along the *c* axis (Fig. 3). The crystal is stabilized by intramolecular N—H···O hydrogen bond, weak C—H···O interactions (Table 1) and C—H···π interactions (Table 1); *Cg*1 is the centroid of the C1–C6 ring.

### Experimental

The title compound was obtained by refluxing ethyl 2-hydrazinyl-5-nitrobenzoate (0.01 mol) and 4-(dimethylamino) benzaldehyde (0.01 mol) in ethanol (40 ml) by adding 3 drops of concentrated sulfuric acid for 8 hrs. Excess ethanol was removed from the reaction mixture under reduced pressure. The solid product obtained was filtered, washed with water and dried. Red single crystals of the title compound suitable for *x*-ray structure determination were grown by slow evaporation of an ethanol solution at room temperature (m.p. 439 K).

### Refinement

H atom attached to N atom was located in a difference map and refined isotropically. The remaining H atoms were constrained in a riding motion approximation, with C_aryl_—H = 0.93, C_methylene_—H = 0.97 and C_methyl_—H = 0.96 Å. The *U*_iso_(H) values were constrained to be 1.5*U*_eq_ of the carrier atom for methyl H atoms and 1.2*U*_eq_ for the remaining H atoms. A rotating group model was used for the methyl groups. The highest residual electron density peak is located at 0.70 Å from C1 and the deepest hole is located at 0.64 Å from N4.

### Crystal data

**Table:**

C_18_H_20_N_4_O_4_	*F*_000_ = 752
*M**_r_* = 356.38	*D*_x_ = 1.381 Mg m^−^^3^
Monoclinic, *P*2_1_/*c*	Melting point: 439 K
Hall symbol: -P 2ybc	Mo *K*α radiation λ = 0.71073 Å
*a* = 10.8216 (4) Å	Cell parameters from 3929 reflections
*b* = 15.9175 (6) Å	θ = 2.0–27.5º
*c* = 10.4136 (4) Å	µ = 0.10 mm^−^^1^
β = 107.091 (2)º	*T* = 100.0 (1) K
*V* = 1714.56 (11) Å^3^	Block, red
*Z* = 4	0.44 × 0.41 × 0.31 mm

### Data collection

**Table:**

Bruker SMART APEXII CCD area-detector diffractometer	3929 independent reflections
Radiation source: fine-focus sealed tube	3275 reflections with *I* > 2σ(*I*)
Monochromator: graphite	*R*_int_ = 0.029
Detector resolution: 8.33 pixels mm^-1^	θ_max_ = 27.5º
*T* = 100.0(1) K	θ_min_ = 2.0º
ω scans	*h* = −14→12
Absorption correction: multi-scan(*SADABS*; Bruker, 2005)	*k* = −20→19
*T*_min_ = 0.957, *T*_max_ = 0.970	*l* = −13→13
16368 measured reflections	

### Refinement

**Table:**

Refinement on *F*^2^	Secondary atom site location: difference Fourier map
Least-squares matrix: full	Hydrogen site location: inferred from neighbouring sites
*R*[*F*^2^ > 2σ(*F*^2^)] = 0.038	H atoms treated by a mixture of independent and constrained refinement
*wR*(*F*^2^) = 0.105	*w* = 1/[σ^2^(*F*_o_^2^) + (0.0493*P*)^2^ + 0.6489*P*] where *P* = (*F*_o_^2^ + 2*F*_c_^2^)/3
*S* = 1.04	(Δ/σ)_max_ = 0.001
3929 reflections	Δρ_max_ = 0.26 e Å^−^^3^
242 parameters	Δρ_min_ = −0.27 e Å^−^^3^
Primary atom site location: structure-invariant direct methods	Extinction correction: none

### Special details

**Table:**

Experimental. The low-temperature data was collected with the Oxford Cyrosystem Cobra low-temperature attachment.
Geometry. All e.s.d.'s (except the e.s.d. in the dihedral angle between two l.s. planes) are estimated using the full covariance matrix. The cell e.s.d.'s are taken into account individually in the estimation of e.s.d.'s in distances, angles and torsion angles; correlations between e.s.d.'s in cell parameters are only used when they are defined by crystal symmetry. An approximate (isotropic) treatment of cell e.s.d.'s is used for estimating e.s.d.'s involving l.s. planes.
Refinement. Refinement of *F*^2^ against ALL reflections. The weighted *R*-factor *wR* and goodness of fit *S* are based on *F*^2^, conventional *R*-factors *R* are based on *F*, with *F* set to zero for negative *F*^2^. The threshold expression of *F*^2^ > σ(*F*^2^) is used only for calculating *R*-factors(gt) *etc*. and is not relevant to the choice of reflections for refinement. *R*-factors based on *F*^2^ are statistically about twice as large as those based on *F*, and *R*- factors based on ALL data will be even larger.

### Fractional atomic coordinates and isotropic or equivalent isotropic displacement parameters (Å^2^)

**Table:**

	*x*	*y*	*z*	*U*_iso_*/*U*_eq_	
O1	0.79559 (10)	0.52119 (6)	−0.07387 (11)	0.0354 (3)	
O2	0.69063 (9)	0.41140 (6)	−0.17045 (10)	0.0304 (2)	
O3	0.81387 (8)	0.14041 (5)	−0.00146 (9)	0.0220 (2)	
O4	0.93830 (9)	0.11554 (6)	0.20957 (9)	0.0263 (2)	
N1	1.11000 (9)	0.28501 (7)	0.47925 (10)	0.0205 (2)	
N2	1.03176 (10)	0.24883 (7)	0.36376 (10)	0.0200 (2)	
N3	1.51942 (11)	0.31448 (7)	1.07252 (11)	0.0251 (3)	
N4	0.77246 (10)	0.44519 (7)	−0.07681 (11)	0.0233 (2)	
C1	1.27580 (12)	0.33820 (8)	0.74183 (12)	0.0211 (3)	
H1A	1.2308	0.3810	0.6868	0.025*	
C2	1.36107 (12)	0.35818 (8)	0.86484 (12)	0.0217 (3)	
H2A	1.3724	0.4141	0.8915	0.026*	
C3	1.43165 (11)	0.29502 (8)	0.95126 (12)	0.0199 (3)	
C4	1.40814 (11)	0.21103 (8)	0.90855 (12)	0.0205 (3)	
H4A	1.4511	0.1679	0.9641	0.025*	
C5	1.32196 (11)	0.19195 (8)	0.78513 (12)	0.0204 (3)	
H5A	1.3078	0.1360	0.7594	0.024*	
C6	1.25546 (11)	0.25480 (8)	0.69791 (12)	0.0190 (3)	
C7	1.16926 (11)	0.23101 (8)	0.56787 (12)	0.0200 (3)	
H7A	1.1563	0.1742	0.5477	0.024*	
C8	0.97180 (11)	0.29570 (8)	0.25563 (12)	0.0186 (2)	
C9	0.89357 (11)	0.25738 (8)	0.13494 (12)	0.0185 (2)	
C10	0.82866 (11)	0.30810 (8)	0.02772 (12)	0.0188 (2)	
H10A	0.7750	0.2839	−0.0500	0.023*	
C11	0.84340 (11)	0.39425 (8)	0.03584 (12)	0.0200 (3)	
C12	0.92453 (12)	0.43263 (8)	0.15043 (13)	0.0227 (3)	
H12A	0.9362	0.4906	0.1531	0.027*	
C13	0.98667 (12)	0.38414 (8)	0.25861 (13)	0.0219 (3)	
H13A	1.0396	0.4097	0.3355	0.026*	
C14	0.88536 (11)	0.16499 (8)	0.12145 (12)	0.0197 (3)	
C15	0.80366 (13)	0.05029 (8)	−0.02550 (13)	0.0235 (3)	
H15A	0.8831	0.0233	0.0262	0.028*	
H15B	0.7926	0.0394	−0.1199	0.028*	
C16	0.69201 (16)	0.01346 (9)	0.01292 (16)	0.0362 (4)	
H16A	0.6876	−0.0459	−0.0045	0.054*	
H16B	0.6132	0.0396	−0.0389	0.054*	
H16C	0.7038	0.0230	0.1068	0.054*	
C17	1.57956 (12)	0.24807 (9)	1.16534 (13)	0.0243 (3)	
H17A	1.6259	0.2111	1.1230	0.036*	
H17B	1.5141	0.2169	1.1902	0.036*	
H17C	1.6385	0.2722	1.2442	0.036*	
C18	1.53899 (13)	0.40099 (8)	1.11646 (13)	0.0276 (3)	
H18A	1.5651	0.4334	1.0511	0.041*	
H18B	1.6051	0.4037	1.2012	0.041*	
H18C	1.4598	0.4233	1.1262	0.041*	
H1N2	1.0273 (15)	0.1942 (11)	0.3542 (16)	0.034 (4)*	

### Atomic displacement parameters (Å^2^)

**Table:**

	*U*^11^	*U*^22^	*U*^33^	*U*^12^	*U*^13^	*U*^23^
O1	0.0480 (6)	0.0165 (5)	0.0349 (6)	−0.0003 (4)	0.0017 (5)	0.0046 (4)
O2	0.0372 (5)	0.0261 (5)	0.0215 (5)	−0.0018 (4)	−0.0011 (4)	0.0025 (4)
O3	0.0290 (4)	0.0158 (4)	0.0178 (4)	−0.0011 (3)	0.0017 (4)	−0.0012 (3)
O4	0.0334 (5)	0.0205 (5)	0.0201 (5)	−0.0013 (4)	−0.0001 (4)	0.0025 (4)
N1	0.0205 (5)	0.0251 (5)	0.0149 (5)	−0.0033 (4)	0.0039 (4)	−0.0025 (4)
N2	0.0239 (5)	0.0194 (5)	0.0152 (5)	−0.0029 (4)	0.0036 (4)	−0.0024 (4)
N3	0.0307 (6)	0.0223 (6)	0.0174 (5)	−0.0018 (4)	−0.0006 (4)	0.0005 (4)
N4	0.0282 (5)	0.0195 (5)	0.0221 (6)	0.0014 (4)	0.0070 (4)	0.0018 (4)
C1	0.0239 (6)	0.0203 (6)	0.0180 (6)	0.0012 (5)	0.0043 (5)	0.0027 (5)
C2	0.0273 (6)	0.0167 (6)	0.0202 (6)	−0.0015 (5)	0.0057 (5)	−0.0005 (5)
C3	0.0220 (6)	0.0222 (6)	0.0150 (6)	−0.0018 (5)	0.0047 (5)	0.0006 (5)
C4	0.0233 (6)	0.0196 (6)	0.0182 (6)	0.0015 (4)	0.0054 (5)	0.0038 (5)
C5	0.0239 (6)	0.0181 (6)	0.0198 (6)	−0.0018 (5)	0.0075 (5)	−0.0005 (5)
C6	0.0199 (5)	0.0214 (6)	0.0163 (6)	−0.0017 (4)	0.0060 (5)	−0.0002 (5)
C7	0.0221 (5)	0.0198 (6)	0.0190 (6)	−0.0024 (5)	0.0076 (5)	−0.0019 (5)
C8	0.0186 (5)	0.0216 (6)	0.0166 (6)	−0.0004 (4)	0.0065 (5)	−0.0006 (5)
C9	0.0198 (5)	0.0188 (6)	0.0172 (6)	−0.0013 (4)	0.0059 (5)	−0.0006 (5)
C10	0.0215 (5)	0.0196 (6)	0.0154 (6)	−0.0015 (4)	0.0054 (5)	−0.0015 (5)
C11	0.0228 (6)	0.0194 (6)	0.0179 (6)	0.0014 (4)	0.0063 (5)	0.0026 (5)
C12	0.0270 (6)	0.0171 (6)	0.0244 (7)	−0.0019 (5)	0.0081 (5)	−0.0026 (5)
C13	0.0246 (6)	0.0218 (6)	0.0185 (6)	−0.0023 (5)	0.0050 (5)	−0.0045 (5)
C14	0.0204 (5)	0.0206 (6)	0.0172 (6)	−0.0017 (4)	0.0043 (5)	−0.0012 (5)
C15	0.0338 (7)	0.0145 (6)	0.0197 (6)	0.0004 (5)	0.0042 (5)	−0.0025 (5)
C16	0.0544 (9)	0.0263 (7)	0.0337 (8)	−0.0135 (6)	0.0218 (7)	−0.0090 (6)
C17	0.0249 (6)	0.0279 (7)	0.0172 (6)	0.0014 (5)	0.0018 (5)	0.0031 (5)
C18	0.0311 (7)	0.0266 (7)	0.0199 (7)	−0.0030 (5)	−0.0003 (5)	−0.0031 (5)

### Geometric parameters (Å, °)

**Table:**

O1—N4	1.2340 (14)	C7—H7A	0.9300
O2—N4	1.2316 (14)	C8—C13	1.4162 (17)
O3—C14	1.3445 (14)	C8—C9	1.4294 (16)
O3—C15	1.4548 (14)	C9—C10	1.3896 (17)
O4—C14	1.2170 (15)	C9—C14	1.4775 (17)
N1—C7	1.2861 (16)	C10—C11	1.3803 (17)
N1—N2	1.3770 (14)	C10—H10A	0.9300
N2—C8	1.3476 (16)	C11—C12	1.3973 (17)
N2—H1N2	0.874 (17)	C12—C13	1.3682 (18)
N3—C3	1.3739 (15)	C12—H12A	0.9300
N3—C18	1.4469 (17)	C13—H13A	0.9300
N3—C17	1.4511 (16)	C15—C16	1.499 (2)
N4—C11	1.4469 (16)	C15—H15A	0.9700
C1—C2	1.3779 (17)	C15—H15B	0.9700
C1—C6	1.4003 (17)	C16—H16A	0.9600
C1—H1A	0.9300	C16—H16B	0.9600
C2—C3	1.4141 (17)	C16—H16C	0.9600
C2—H2A	0.9300	C17—H17A	0.9600
C3—C4	1.4084 (17)	C17—H17B	0.9600
C4—C5	1.3817 (17)	C17—H17C	0.9600
C4—H4A	0.9300	C18—H18A	0.9600
C5—C6	1.3999 (17)	C18—H18B	0.9600
C5—H5A	0.9300	C18—H18C	0.9600
C6—C7	1.4509 (16)		
			
C14—O3—C15	116.37 (9)	C11—C10—H10A	119.8
C7—N1—N2	113.34 (10)	C9—C10—H10A	119.8
C8—N2—N1	121.31 (10)	C10—C11—C12	121.27 (11)
C8—N2—H1N2	117.3 (11)	C10—C11—N4	118.87 (11)
N1—N2—H1N2	120.9 (11)	C12—C11—N4	119.86 (11)
C3—N3—C18	120.17 (11)	C13—C12—C11	119.34 (12)
C3—N3—C17	120.11 (11)	C13—C12—H12A	120.3
C18—N3—C17	119.16 (10)	C11—C12—H12A	120.3
O2—N4—O1	122.76 (11)	C12—C13—C8	121.17 (11)
O2—N4—C11	118.95 (10)	C12—C13—H13A	119.4
O1—N4—C11	118.29 (11)	C8—C13—H13A	119.4
C2—C1—C6	121.36 (11)	O4—C14—O3	122.77 (11)
C2—C1—H1A	119.3	O4—C14—C9	124.77 (11)
C6—C1—H1A	119.3	O3—C14—C9	112.45 (10)
C1—C2—C3	121.09 (12)	O3—C15—C16	111.47 (11)
C1—C2—H2A	119.5	O3—C15—H15A	109.3
C3—C2—H2A	119.5	C16—C15—H15A	109.3
N3—C3—C4	121.08 (11)	O3—C15—H15B	109.3
N3—C3—C2	121.52 (11)	C16—C15—H15B	109.3
C4—C3—C2	117.40 (11)	H15A—C15—H15B	108.0
C5—C4—C3	120.82 (11)	C15—C16—H16A	109.5
C5—C4—H4A	119.6	C15—C16—H16B	109.5
C3—C4—H4A	119.6	H16A—C16—H16B	109.5
C4—C5—C6	121.61 (11)	C15—C16—H16C	109.5
C4—C5—H5A	119.2	H16A—C16—H16C	109.5
C6—C5—H5A	119.2	H16B—C16—H16C	109.5
C5—C6—C1	117.65 (11)	N3—C17—H17A	109.5
C5—C6—C7	119.07 (11)	N3—C17—H17B	109.5
C1—C6—C7	123.27 (11)	H17A—C17—H17B	109.5
N1—C7—C6	122.92 (11)	N3—C17—H17C	109.5
N1—C7—H7A	118.5	H17A—C17—H17C	109.5
C6—C7—H7A	118.5	H17B—C17—H17C	109.5
N2—C8—C13	120.55 (11)	N3—C18—H18A	109.5
N2—C8—C9	120.92 (11)	N3—C18—H18B	109.5
C13—C8—C9	118.53 (11)	H18A—C18—H18B	109.5
C10—C9—C8	119.18 (11)	N3—C18—H18C	109.5
C10—C9—C14	119.99 (11)	H18A—C18—H18C	109.5
C8—C9—C14	120.79 (11)	H18B—C18—H18C	109.5
C11—C10—C9	120.39 (11)		
			
C7—N1—N2—C8	−173.78 (11)	N2—C8—C9—C14	−5.29 (17)
C6—C1—C2—C3	0.22 (19)	C13—C8—C9—C14	173.76 (11)
C18—N3—C3—C4	−177.96 (12)	C8—C9—C10—C11	2.42 (17)
C17—N3—C3—C4	−6.57 (18)	C14—C9—C10—C11	−175.19 (11)
C18—N3—C3—C2	1.74 (19)	C9—C10—C11—C12	0.79 (18)
C17—N3—C3—C2	173.13 (12)	C9—C10—C11—N4	−178.88 (11)
C1—C2—C3—N3	178.24 (12)	O2—N4—C11—C10	7.77 (17)
C1—C2—C3—C4	−2.05 (18)	O1—N4—C11—C10	−173.19 (12)
N3—C3—C4—C5	−178.50 (11)	O2—N4—C11—C12	−171.90 (11)
C2—C3—C4—C5	1.79 (18)	O1—N4—C11—C12	7.14 (18)
C3—C4—C5—C6	0.30 (19)	C10—C11—C12—C13	−2.54 (19)
C4—C5—C6—C1	−2.13 (18)	N4—C11—C12—C13	177.12 (11)
C4—C5—C6—C7	178.08 (11)	C11—C12—C13—C8	1.02 (19)
C2—C1—C6—C5	1.87 (18)	N2—C8—C13—C12	−178.82 (11)
C2—C1—C6—C7	−178.35 (12)	C9—C8—C13—C12	2.13 (18)
N2—N1—C7—C6	−179.18 (10)	C15—O3—C14—O4	−0.73 (17)
C5—C6—C7—N1	−176.20 (11)	C15—O3—C14—C9	178.13 (10)
C1—C6—C7—N1	4.02 (19)	C10—C9—C14—O4	−179.76 (12)
N1—N2—C8—C13	−0.74 (17)	C8—C9—C14—O4	2.67 (19)
N1—N2—C8—C9	178.29 (10)	C10—C9—C14—O3	1.40 (16)
N2—C8—C9—C10	177.12 (11)	C8—C9—C14—O3	−176.17 (10)
C13—C8—C9—C10	−3.83 (17)	C14—O3—C15—C16	88.66 (13)

### Hydrogen-bond geometry (Å, °)

**Table:**

*D*—H···*A*	*D*—H	H···*A*	*D*···*A*	*D*—H···*A*
N2—H1N2···O4	0.875 (18)	1.978 (17)	2.6736 (14)	135.6 (14)
C7—H7A···O1^i^	0.93	2.49	3.3599 (16)	156
C12—H12A···O4^ii^	0.93	2.59	3.3961 (16)	145
C16—H16C···O2^iii^	0.96	2.59	3.5116 (19)	162
C17—H17B···Cg1^iii^	0.96	2.64	3.4629 (14)	144

Symmetry codes:  (i) −*x*+2, *y*−1/2, −*z*+1/2; (ii) −*x*+2, *y*+1/2, −*z*+1/2; (iii) *x*, −*y*+1/2, *z*+1/2.
